# Repetitive Head Impacts and Perivascular Space Volume in Former American Football Players

**DOI:** 10.1001/jamanetworkopen.2024.28687

**Published:** 2024-08-01

**Authors:** Leonard B. Jung, Tim L. T. Wiegand, Fatima Tuz-Zahra, Yorghos Tripodis, Jeffrey J. Iliff, Juan Piantino, Hector Arciniega, Cara L. Kim, Lara Pankatz, Sylvain Bouix, Alexander P. Lin, Michael L. Alosco, Daniel H. Daneshvar, Jesse Mez, Farshid Sepehrband, Yogesh Rathi, Ofer Pasternak, Michael J. Coleman, Charles H. Adler, Charles Bernick, Laura Balcer, Jeffrey L. Cummings, Eric M. Reiman, Robert A. Stern, Martha E. Shenton, Inga K. Koerte

**Affiliations:** Psychiatry Neuroimaging Laboratory, Department of Psychiatry, Brigham and Women’s Hospital, Harvard Medical School, Boston, Massachusetts; cBRAIN, Department of Child and Adolescent Psychiatry, Psychosomatics, and Psychotherapy, Ludwig-Maximilians-Universität, Munich, Germany.; Psychiatry Neuroimaging Laboratory, Department of Psychiatry, Brigham and Women’s Hospital, Harvard Medical School, Boston, Massachusetts; cBRAIN, Department of Child and Adolescent Psychiatry, Psychosomatics, and Psychotherapy, Ludwig-Maximilians-Universität, Munich, Germany.; Department of Biostatistics, Boston University School of Public Health, Boston, Massachusetts; Department of Biostatistics, Boston University School of Public Health, Boston, Massachusetts; Boston University Alzheimer’s Disease Research Center, Boston University CTE Center, Boston University School of Medicine, Boston, Massachusetts; Department of Psychiatry and Behavioral Sciences, University of Washington School of Medicine, Seattle; Department of Neurology, University of Washington School of Medicine, Seattle; VISN 20 Northwest Network Mental Illness Research, Education and Clinical Center, VA Puget Sound Health Care System, Seattle, Washington; Department of Pediatrics, Division of Child Neurology, Doernbecher Children’s Hospital, Oregon Health and Science University, Portland; Psychiatry Neuroimaging Laboratory, Department of Psychiatry, Brigham and Women’s Hospital, Harvard Medical School, Boston, Massachusetts; Department of Rehabilitation Medicine, NYU Grossman School of Medicine, New York, New York; Psychiatry Neuroimaging Laboratory, Department of Psychiatry, Brigham and Women’s Hospital, Harvard Medical School, Boston, Massachusetts; cBRAIN, Department of Child and Adolescent Psychiatry, Psychosomatics, and Psychotherapy, Ludwig-Maximilians-Universität, Munich, Germany.; Psychiatry Neuroimaging Laboratory, Department of Psychiatry, Brigham and Women’s Hospital, Harvard Medical School, Boston, Massachusetts; cBRAIN, Department of Child and Adolescent Psychiatry, Psychosomatics, and Psychotherapy, Ludwig-Maximilians-Universität, Munich, Germany.; Psychiatry Neuroimaging Laboratory, Department of Psychiatry, Brigham and Women’s Hospital, Harvard Medical School, Boston, Massachusetts; Département de génie logiciel et TI, École de technologie supérieure, Université du Québec, Montreal, Canada; Psychiatry Neuroimaging Laboratory, Department of Psychiatry, Brigham and Women’s Hospital, Harvard Medical School, Boston, Massachusetts; Center for Clinical Spectroscopy, Department of Radiology, Brigham and Women’s Hospital, Harvard Medical School, Boston, Massachusetts; Boston University Alzheimer’s Disease Research Center, Boston University CTE Center, Department of Neurology, Boston University School of Medicine, Boston, Massachusetts; Department of Physical Medicine and Rehabilitation, Massachusetts General Hospital, Harvard Medical School, Boston; Boston University Alzheimer’s Disease Research Center, Boston University CTE Center, Department of Neurology, Boston University School of Medicine, Boston, Massachusetts; Stevens Neuroimaging and Informatics Institute, Keck School of Medicine of USC, University of Southern California, Los Angeles; Psychiatry Neuroimaging Laboratory, Department of Psychiatry, Brigham and Women’s Hospital, Harvard Medical School, Boston, Massachusetts; Department of Psychiatry, Massachusetts General Hospital, Harvard Medical School, Boston; Psychiatry Neuroimaging Laboratory, Department of Psychiatry, Brigham and Women’s Hospital, Harvard Medical School, Boston, Massachusetts; Department of Psychiatry, Massachusetts General Hospital, Harvard Medical School, Boston; Department of Radiology, Brigham and Women’s Hospital, Harvard Medical School, Boston, Massachusetts; Psychiatry Neuroimaging Laboratory, Department of Psychiatry, Brigham and Women’s Hospital, Harvard Medical School, Boston, Massachusetts; Department of Neurology, Mayo Clinic College of Medicine, Mayo Clinic Arizona Scottsdale, Arizona; Cleveland Clinic Lou Ruvo Center for Brain Health, Las Vegas, Nevada; Department of Neurology, NYU Grossman School of Medicine, New York, New York; Department of Population Health, NYU Grossman School of Medicine, New York, New York; Department of Ophthalmology, NYU Grossman School of Medicine, New York, New York; Chambers-Grundy Center for Transformative Neuroscience, Pam Quirk Brain Health and Biomarker Laboratory, Department of Brain Health, School of Integrated Health Sciences, University of Nevada, Las Vegas; Banner Alzheimer’s Institute, University of Arizona, Arizona State University, Translational Genomics Research Institute, and Arizona Alzheimer’s Consortium, Phoenix; Boston University Alzheimer’s Disease Research Center, Boston University CTE Center, Department of Neurology, Boston University School of Medicine, Boston, Massachusetts; Department of Anatomy & Neurobiology, Boston University School of Medicine, Boston, Massachusetts; Department of Neurosurgery, Boston University School of Medicine, Boston, Massachusetts; Psychiatry Neuroimaging Laboratory, Department of Psychiatry, Brigham and Women’s Hospital, Harvard Medical School, Boston, Massachusetts; Department of Psychiatry, Massachusetts General Hospital, Harvard Medical School, Boston; Department of Radiology, Brigham and Women’s Hospital, Harvard Medical School, Boston, Massachusetts; Psychiatry Neuroimaging Laboratory, Department of Psychiatry, Brigham and Women’s Hospital, Harvard Medical School, Boston, Massachusetts; cBRAIN, Department of Child and Adolescent Psychiatry, Psychosomatics, and Psychotherapy, Ludwig-Maximilians-Universität, Munich, Germany.; Department of Psychiatry, Massachusetts General Hospital, Harvard Medical School, Boston; Graduate School of Systemic Neurosciences, Ludwig-Maximilians-Universität, Munich, Germany

## Abstract

**IMPORTANCE:**

Exposure to repetitive head impacts (RHI) is associated with increased risk for neurodegeneration. Accumulation of toxic proteins due to impaired brain clearance is suspected to play a role.

**OBJECTIVE:**

To investigate whether perivascular space (PVS) volume is associated with lifetime exposure to RHI in individuals at risk for RHI-associated neurodegeneration.

**DESIGN, SETTING, AND PARTICIPANTS:**

This cross-sectional study was part of the Diagnostics, Imaging, and Genetics Network for the Objective Study and Evaluation of Chronic Traumatic Encephalopathy (DIAGNOSE CTE) Research Project, a 7-year multicenter study consisting of 4 US study sites. Data were collected from September 2016 to February 2020 and analyses were performed between May 2021 and October 2023. After controlling for magnetic resonance image (MRI) and processing quality, former American football players and unexposed asymptomatic control participants were included in analyses.

**EXPOSURE:**

Prior exposure to RHI while participating in American football was estimated using the 3 cumulative head impact indices (CHII-G, linear acceleration; CHII-R, rotational acceleration; and CHII, number of head impacts).

**MAIN OUTCOMES AND MEASURES:**

Individual PVS volume was calculated in the white matter of structural MRI. Cognitive impairment was based on neuropsychological assessment. Linear regression models were used to assess associations of PVS volume with neuropsychological assessments in former American football players. All analyses were adjusted for confounders associated with PVS volume.

**RESULTS:**

Analyses included 224 participants (median [IQR] age, 57 [51–65] years), with 170 male former football players (114 former professional athletes, 56 former collegiate athletes) and 54 male unexposed control participants. Former football players had larger PVS volume compared with the unexposed group (mean difference, 0.28 [95% CI, 0.00–0.56]; *P* = .05). Within the football group, PVS volume was associated with higher CHII-R (β = 2.71 × 10^−8^ [95% CI, 0.50 × 10^−8^ to 4.93 × 10^−8^]; *P* = .03) and CHII-G (β = 2.24 × 10^−6^ [95% CI, 0.35 × 10^−6^ to 4.13 × 10^−6^]; *P* = .03). Larger PVS volume was also associated with worse performance on cognitive functioning in former American football players (β = −0.74 [95% CI, −1.35 to −0.13]; *P* = .04).

**CONCLUSIONS AND RELEVANCE:**

These findings suggest that impaired perivascular brain clearance, as indicated by larger PVS volume, may contribute to the association observed between RHI exposure and neurodegeneration.

## Introduction

Repetitive head impacts (RHI) affect millions of people worldwide every day while participating in contact sports.^1,2^ An evolving body of evidence suggests an association between RHI exposure and an increased likelihood of developing neurodegenerative disorders and dementia later in life.^3–8^ The exact pathophysiological processes leading to neurodegeneration are unknown.^9^ However, postmortem studies demonstrate that progressive dementia following RHI exposure is associated with an accumulation of tau proteins in the brain.^6,10,11^ Thus, there is a need to improve our understanding of why proteins accumulate in the brain following exposure to RHI.^12^

Perivascular transport is considered responsible for clearing proteins from the brain.^13–16^ Arterial pulsations drive cerebrospinal fluid (CSF) from the subarachnoid space toward the brain parenchyma along the perivascular space (PVS) of brain-penetrating arterioles.^17,18^ There, brain waste products are cleared from the brain parenchyma along the PVS surrounding ascending veins.^19^ While the precise pathways for fluid exit remain under investigation, it is hypothesized that following the clearance of the brain parenchyma, cerebral waste is partially transported via meningeal lymphatic vessels before finally draining into cervical lymph nodes.^19–22^ Of note, perivascular transport, which may also include a glial component (then called *glymphatic transport* and involving aquaporin 4 water channels),^23,24^ is reported to be involved in clearing toxic proteins from the brain in Alzheimer disease^16^ and also following severe traumatic brain injury (TBI).^15,25–27^ However, to date, it is not known whether exposure to RHI affects either perivascular transport or structures of the perivascular transport system. If it does, then it may contribute to neurodegenerative processes and ultimately to cognitive decline in individuals exposed to RHI.

Usually microscopic in size, PVS can become enlarged and thereby visible on structural magnetic resonance imaging (MRI).^28–30^ While not directly portraying cerebral fluid dynamics, enlarged PVS is considered a potential structural imaging marker indicative of impaired perivascular transport.^31,32^ Previous research suggests that the presence of MRI-visible PVS is associated with an increased risk of developing neurodegenerative diseases in general that could lead to cognitive decline and dementia over time. More specifically, PVS has been found to be associated with increased odds of vascular dementia based on a 4-year follow-up in a sample of more than 2600 MRI scans.^33^ Another study reported PVS to be associated with higher odds of dementia over a period of 8 years based on 1400 MRI scans from the Framingham Heart Study.^34^ However, a meta-analysis by Hilal et al^35^ did not find an association between PVS and mild cognitive impairment across a sample of more than 3000 MRI scans. Additionally, a study by Sim et al^36^ did not find an association between the degree of hippocampal PVS and memory function in 109 older participants without dementia.^36^ Of note, these latter analyses did not perform longitudinal analyses. More detailed information regarding the use of MRI to visualize PVS is included in eAppendix 1 in Supplement 1.

The aim of this study is to investigate whether PVS volume, as measured on structural MRI, is associated with RHI exposure in individuals with a history of extensive exposure to RHI while playing American football. We further investigate whether PVS volume is associated with neuropsychological functioning (eg, measures of general cognitive functioning, memory, and executive functioning).

## Methods

This cross-sectional study and its procedures were granted approval by the Boston University Medical Campus, Cleveland Clinic Lou Ruvo Center for Brain Health, Mayo Clinic and Banner Alzheimer Institute, New York University Medical Center Langone, and Partners Healthcare–Brigham and Women’s Hospital institutional review boards. All participants provided written informed consent prior to enrollment.^37^ This study is reported following the Strengthening the Reporting of Observational Studies in Epidemiology (STROBE) reporting guideline.

### Study Design and Participants

This study is part of the Diagnostics, Imaging, And Genetics Network for the Objective Study and Evaluation of Chronic Traumatic Encephalopathy (DIAGNOSE CTE) Research Project aiming to develop biomarkers for the in vivo diagnosis of CTE.^37^ Participant recruitment was performed with social media posts and newspaper and billboard advertisements. Former football players were additionally informed about the DIAGNOSE CTE study via videos of former Super Bowl champion Ben Utecht and by direct community outreach via National Football League alumni groups. Further details on recruitment have been published elsewhere.^37^ The study includes a neuropsychological test battery, an evaluation of exposure to RHI, a neurologic examination, neuropsychiatric questionnaires, fluid biomarkers, and neuroimaging. Neuroimaging includes multisequence MRI, diffusion MRI, magnetic resonance spectroscopy, and positron emission tomography. The study population comprises former professional American football players, former collegiate football players, and a group of same-age men without exposure to RHI (unexposed comparison group).

The DIAGNOSE CTE Research Project enrollment criteria were divided into general inclusion and exclusion criteria applied to all 3 groups, as well as group-specific criteria. Data were collected from September 2016 to February 2020. General inclusion criteria were male sex, age 45 to 74 years, no contraindications for lumbar puncture or MRI or positron emission tomography scans, English as primary language, agreement to all procedures, and availability of a study partner (ie, a spouse or family member). General exclusion criteria were a history of significant neurologic condition, vision or hearing impairment hindering neuropsychological testing, impaired decision capacity to consent to participation, or significant comorbidities (ie, infectious, endocrine, or metabolic disease; pulmonary, kidney, or liver impairment; cancer; body weight >400 lbs). Specific inclusion criteria for former professional football players were minimum of 12 years of organized football play at high head impact playing positions (ie, not kicker or quarterback) with at least 3 seasons at the college level and 4 seasons in the National Football League. Similar guidelines applied to the collegiate athletes, although a minimum of 6 years of organized football play with at least 3 years at the college level and no organized football or contact sports, thereafter, were required. Enrollment criteria for participants in the unexposed comparison group were no history of TBI or concussion; no participation in organized contact sports or military combat service or training; no formal diagnosis or treatment needed for psychiatric illness or cognitive impairment; asymptomatic on telephone screening regarding mood, behavior, cognitive symptoms and functional independence; a minimum of 2 years of postsecondary education or an associate’s degree; and a minimum body mass index (BMI; calculated as weight in kilograms divided by height in meters squared) of 24. All participants provided a detailed medical history, including medical diagnoses and medications. Details regarding the medication questionnaires used to gain information about medications are described in eAppendix 2 in Supplement 1.

In total, 180 former football players were included in the DIAGNOSE CTE Research Project (120 former professional players and 60 former collegiate athletes). For the present analyses, data from 10 former football players were excluded (6 with missing T1-weighted [T1w] images; 1 with T1w images failed to process; 1 with T2-weighted [T2w] images missing; 1 with T2w images scanned using another MRI scanner with different sequence parameters; 1 with failed PVS processing ). This left a sample of 170 former football players. There were 60 unexposed participants included in the overall project, of which data from 6 participants were excluded (1 with missing T1w images; 1 with missing T2w images; 3 with undisclosed exposure to RHI; 1 with undisclosed long-standing psychiatric disorder), resulting in 54 unexposed participants.

### Exposure Variables

To estimate the cumulative head impact exposure for each participant, 3 previously described exposure measures were calculated cumulative number of impacts (CHII), cumulative linear acceleration (g-force; CHII-G), and cumulative rotational acceleration (radians/s^2^; CHII-R). These calculations use self-reported information on the number of American football seasons played at each level and on-field playing position at each level. The latter were in addition to helmet accelerometer data (mean head impact frequencies, mean linear accelerations, and mean rotational accelerations) by playing position and level of play, as described elsewhere.^11,38,39^ While the 3 CHII scores share a high degree of collinearity due to their common basis in years of play and playing level, they are distinguished by the head impact acceleration characteristics associated with each American football playing position at each level.^11^ For instance, offensive linemen experience a high frequency of lower magnitude head impacts, whereas wide receivers encounter fewer but more intense head impacts. Similar differences in playing position are seen when comparing rotational and linear forces. This distinction allows for the variability observed among CHII, CHII-G, and CHII-R, despite their shared foundations. These differences may reflect different biomechanical pathways between head impacts and observed outcomes. Higher CHII scores reflect greater exposure to RHI in the respective head impact domain (head impact frequency, linear acceleration, and rotational acceleration).

### Neuropsychological and Neuropsychiatric Testing

All study participants completed an extensive neuropsychological test battery to evaluate cognitive functioning.^37^ For this study, we opted for the use of raw scores rather than adjusted T-scores. This decision was made to enhance the interpretability of β coefficients in widely recognized neuropsychological assessments such as the Montreal Cognitive Assessment (MoCA) and Trail Making Tests. Consequently, this approach allowed us to adjust for age and education directly in our models without being concerned about double-correcting for these variables. Raw scores of the following tests were selected a priori to address study hypotheses and to reduce the number of variables: MoCA, a measure of general cognitive status^40^; Neuropsychological Assessment Battery (NAB) List Learning Test long delay recall, a measure of episodic memory^41^; and Trail Making Test Parts A and B and Golden Stroop Color-Word Interference, measures of executive functioning.^42,43^ To broaden the scope of this study, post hoc exploratory analyses between PVS and neuropsychological assessments not primarily linked to symptoms portrayed by former contact sport athletes, including language and visuospatial abilities, are provided in the eTable in Supplement 1. Specifically, we report additional significant associations between larger PVS and worse orientation and worse visuospatial ability. There were no significant associations between PVS and language ability.

### MRI

#### Image Acquisition

Noncontrast brain MRI was conducted at each study site. All images were acquired on a Siemens 3T Magnetom Skyra MRI scanner (Siemens Healthineers), software version VE11, and a 20-channel head coil to fit all sizes. The imaging protocol included a T1w magnetization-prepared rapid gradient echo sequence (TR = 2530 ms; TE = 3.36 ms; T1 = 1100 ms; 7-degree flip angle, 256 FOV; 1 × 1 × 1 mm^3^ voxel size) and a T2w sampling perfection with application-optimized contrasts by using flip-angle evolution image (TR = 3200 ms; TE = 408 ms; 256 FOV; 1 × 1 × 1 mm^3^ voxel size).

#### Image Processing and PVS Analysis

**Figure 1.**
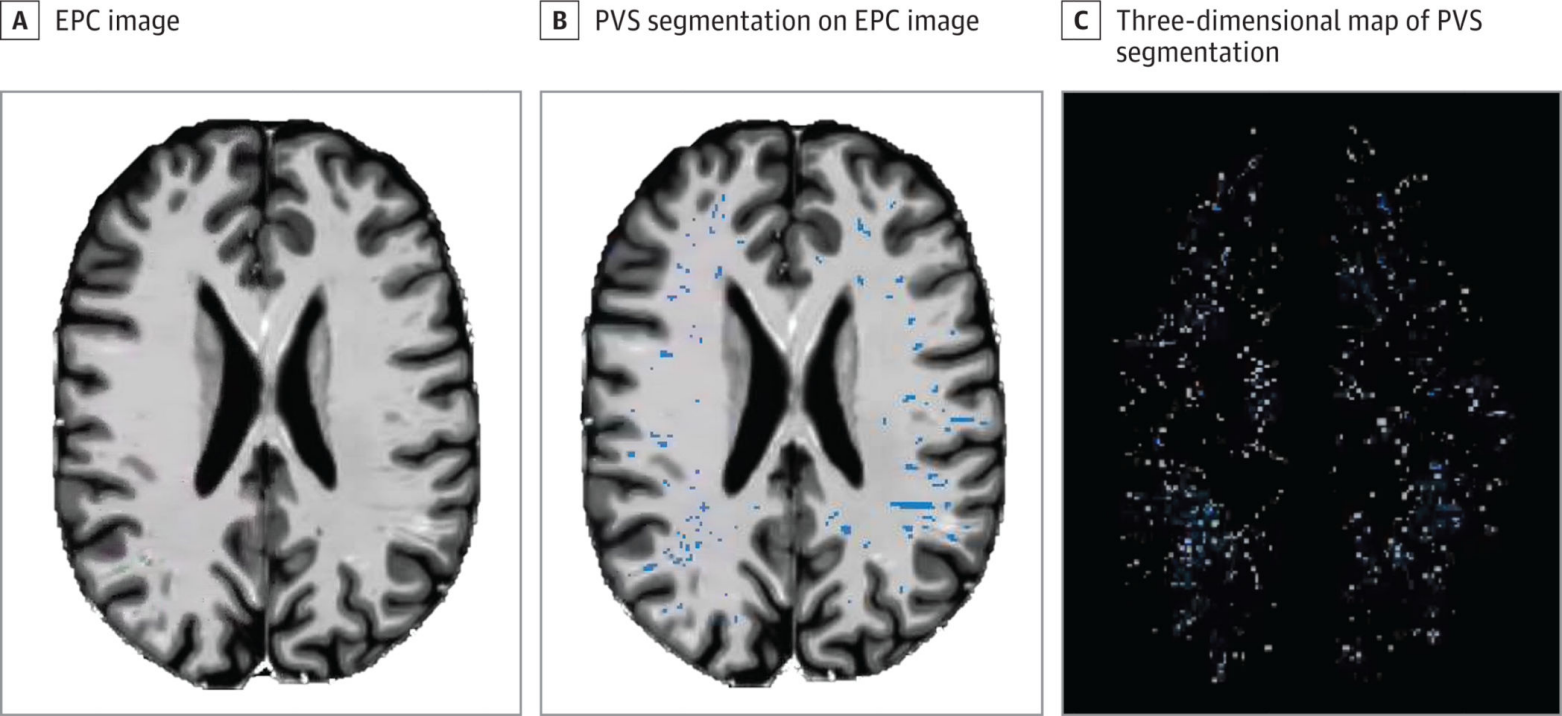
Example of a Perivascular Space (PVS) Segmentation and 3-Dimensional Rendering

Image processing was performed by running the Psychiatry Neuroimaging Laboratory luigi pipeline.^44^ Pipeline steps include quality control, brain masking, and segmentation using FreeSurfer software version 7.1.0 (FreeSurfer). The PVS quantification was performed using a previously published algorithm.^45^ T1w images were divided by T2w images and filtered for tubular structures, producing an enhanced PVS contrast image, from which a white matter (WM) PVS mask was derived. All WM-PVS masks were manually corrected by an experienced, blinded rater (approximately 5 minutes per mask). WM-PVS masks were then used to calculate the individual PVS percentage per WM, ie, the WM-PVS fraction (Figure 1). To approximate statistical normality, the WM-PVS fraction was log-transformed (log_10_) and standardized (log-PVS). To ease readability, log-PVS will be referred to as *PVS volume*. Further details on image processing, PVS analysis, and methodological considerations are described in eAppendix 1 in Supplement 1.

### Statistical Analysis

All statistical analyses were performed using SPSS software version 28.0.0.0 (IBM). *P* values were 2-sided, and *P* ≤ .05 was considered statistically significant. Analyses were performed between May 2021 and October 2023. All analyses were adjusted for the following covariates: age at the time of the MRI scan, BMI, systolic blood pressure, use of antihypertensive medication, and use of blood-brain barrier permeable β-adrenergic receptor blocker (β-blockers; ie, propranolol, metoprolol, nebivolol) specifically, a diagnosis of diabetes, use of antidiabetic medication, a diagnosis of hypercholesterolemia, use of 3-hydroxy-3-methyl-glutaryl-coenzyme A reductase inhibitor (statins), *APOE* ε4-carrier or noncarrier status, years of education, and imaging site. We chose covariates based on evidence of an association with PVS.^46–50^ The specific focus on blood-brain barrier permeable β-blocker intake is based on evidence of adrenergic activity possibly influencing brain clearance.^50,51^ The association between PVS volume and β-blocker intake is discussed further in eAppendix 2 in Supplement 1. The Benjamini-Hochberg false discover rate was used to account for multiple comparisons.

#### Demographics

To assess differences between former football players and the unexposed participants in demographic variables, we performed independent 2-sided Welch *t* test on continuous variables (age at the time of the MRI scan, BMI, years of education, systolic blood pressure) and χ^2^ tests on categorical variables (*APOE* ε4 carrier status, imaging site, self-reported race and ethnicity, antihypertensive medication use, β-blocker use, diagnosis of diabetes, diagnosis of hypercholesterolemia, and statin intake). A generalized linear regression was performed to evaluate the association of demographic variables and log-PVS in the former football player group. Race and ethnicity were classified as American Indian or Alaska Native, Asian, Black or African American, Native Hawaiian or Pacific Islander, White, multiple races, or did not report. Reporting race and ethnicity in this study was mandated by the National Institutes of Health, consistent with Inclusion of Women, Minorities, and Children policy.

#### PVS Volume and RHI

First, a general linear model was applied to compare log-PVS between former football players and the unexposed group. Second, we used multiple general linear regression models to investigate the association of exposure to RHI and log-PVS in the former football player group. CHII scores were the independent variables and log-PVS was the dependent variable.

#### PVS Volume and Cognitive Functioning

We applied multiple generalized linear regression models to evaluate the association between PVS volume and neuropsychological functioning in the former football player group. Specifically, we included log-PVS as the independent variable and the MoCA, NAB List Learning long delay, Trail Making Test A and B, and Golden Stroop color word interference as respective dependent variables.

## Results

### Demographics

The sample demographics are detailed in Table 1. Former football players differed significantly in demographic variables from the unexposed group, with the football players having higher BMI (mean difference, 1.65 [95% CI, 0.21 to 3.11]; *P* = .03) and lower systolic blood pressure (mean difference, 8.14 [95% CI, 4.20 to 12.09]; *P* < .001).

In the former football player group, covariates showed significant associations with log-PVS. Specifically, the older the participant, the larger the log-PVS (β = 0.06 [95% CI, 0.04 to 0.08]; *P* < .001; n = 160). Additionally, former football players using antihypertensive medication showed significantly larger log-PVS (mean difference, 0.34 [95% CI, 0.03 to 0.64]; *P* = .03; n = 160). However, β-blocker intake was significantly associated with lower log-PVS (mean difference, −0.62 [95% CI, −0.14 to −1.10]; *P* = .01; n = 160). The association between antihypertensive medication intake and PVS volume in former American football players is discussed further in eAppendix 2 in Supplement 1.

### PVS Volume and RHI

**Figure 2.**
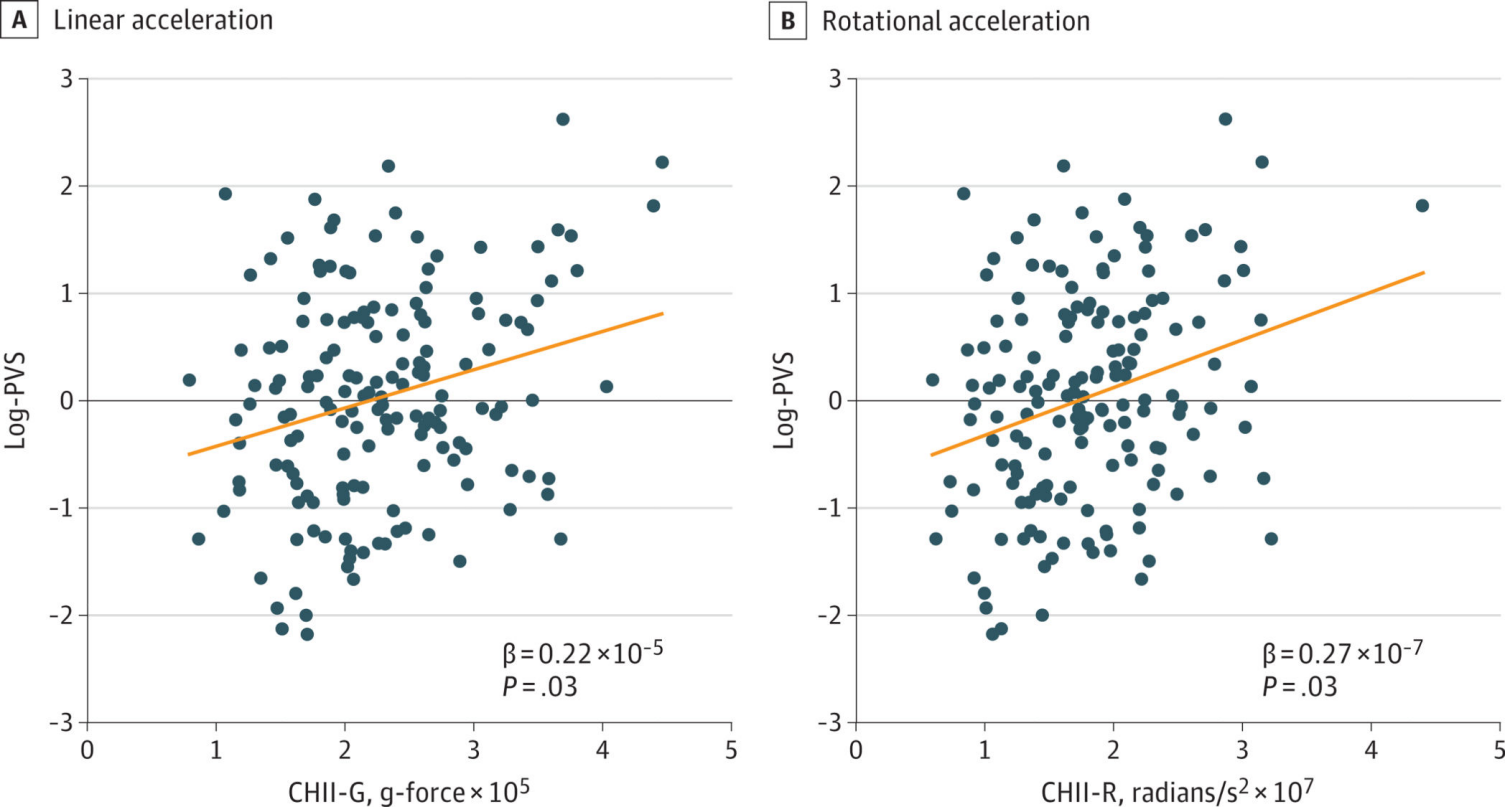
Cumulative Head Impact Indices and Perivascular Space (PVS) Volume Analyses are adjusted for age at the time of magnetic resonance imaging, body mass index, systolic blood pressure, use of antihypertensive medication, and use of blood-brain barrier permeable β-adrenergic receptor blockers (propranolol, metoprolol, nebivolol) intake specifically, diabetes, use of antidiabetic medication, hypercholesterolemia, use of 3-hydroxy-3-methyl-glutaryl-coenzyme A reductase inhibitors intake, *APOE* ε4-carrier vs noncarrier status, years of education, and imaging site. Covariates were chosen based on previous evidence of associations with PVS clearance or head impacts. CHII indicates Cumulative Head Impact Index; G, linear acceleration; R, rotational acceleration. BBB indicates blood-brain barrier; BMI, body mass index.

**Figure 3.**
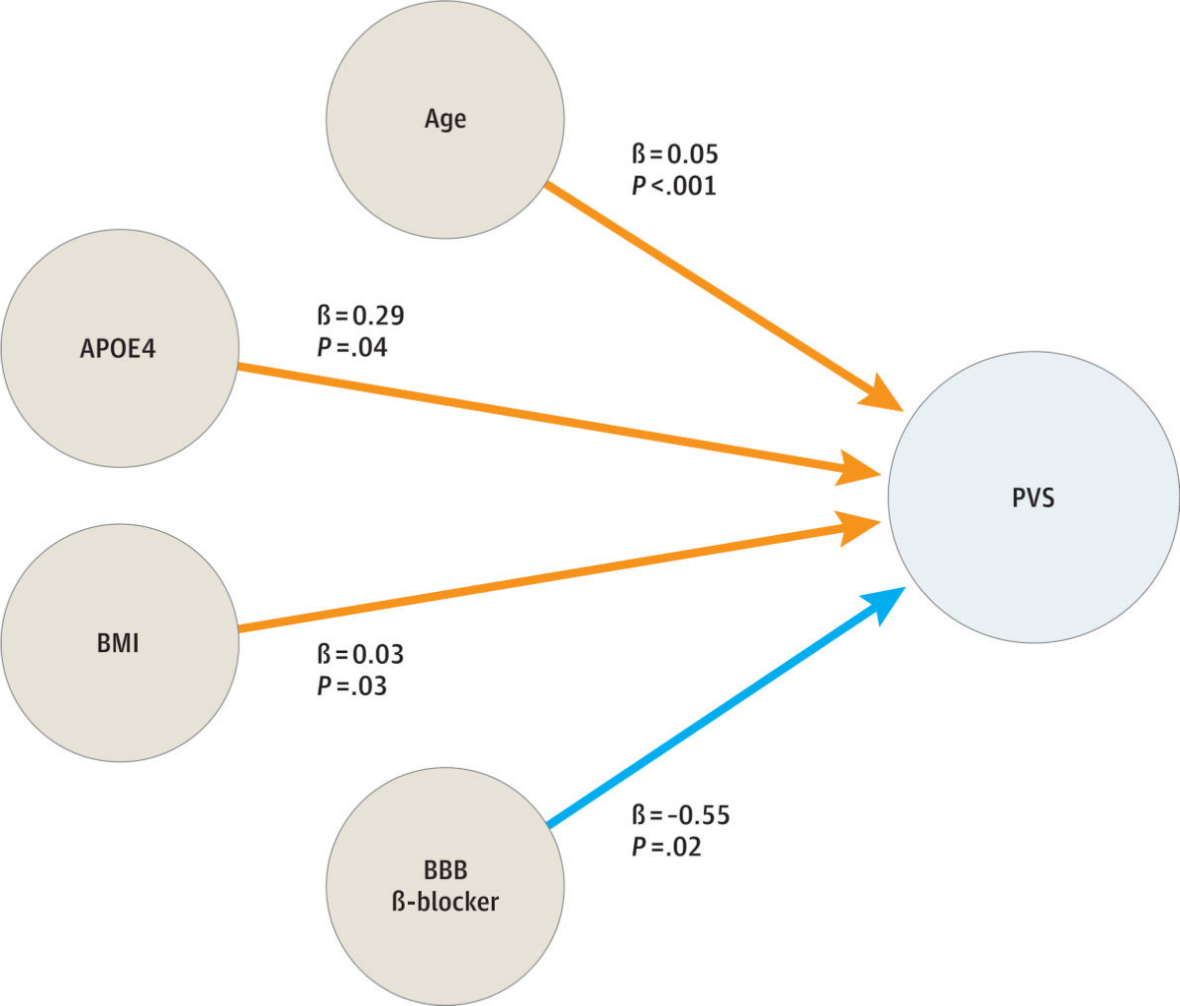
Covariates and Perivascular Space (PVS) in Former American Football Players BBB indicates blood-brain barrier; BMI, body mass index.

Former football players had significantly larger log-PVS than the unexposed group (mean difference, 0.28 [95% CI, 0.00 to 0.76]; *P* = .05; n = 212). In addition, log-PVS was statistically significantly associated with CHII-R (rotational force score, β = 2.71 × 10^−8^ [95% CI, 0.50 × 10^−8^ to 4.93 × 10^−8^]; *P* = .03; n = 160) and CHII-G (linear force score, β = 2.24 × 10^−6^ [95% CI, 0.347 × 10^−6^ to 4.13 × 10^−6^]; *P* = .03; n = 160). This means that an increase of approximately 440 000 g (CHII-G) or 35 million radians/s^2^ (CHII-R) over a career in American football was associated with an increase of 1-SD log-PVS. There was no statistically significant association between log-PVS and the third measure of cumulative head impact exposure, CHII (frequency score, β = 7.55 × 10^−6^ [95% CI, −0.21 × 10^−6^ to 0.36 × 10^−6^]; *P* = .61; n = 160) (Figure 2). The association between CHII-R and log-PVS was significantly influenced by increased age (β = 0.05 [95% CI, 0.04 to 0.07] per 1-year increase; *P* < .001; n = 160), positive *APOE* ε4 carrier status (β = 0.30 [95% CI, 0.02 to 0.58]; *P* = .04; n = 160), and higher BMI (β = 0.03 [95% CI, 0.00 to 0.06]; *P* = .03; n = 160). The association between CHII-G and log-PVS was significantly influenced by increased age (β = 0.05 [95% CI, 0.04 to 0.07] per 1-year increase; *P* < .001; n = 160) and positive *APOE* ε4 carrier status (β = 0.29 [95% CI, 0.01 to 0.56]; *P* = .04; n = 160). Use of β-blockers significantly influenced the association between CHII-R and log-PVS (β = −0.56 [95% CI, −1.04 to −0.09]; *P* = .02; n = 160) as well as CHII-G and log-PVS (β = −0.55 [95% CI, −1.03 to −0.08]; *P* = .02; n = 160). In both models, β-blocker intake was associated with significantly lower log-PVS (Figure 3).

### PVS Volume and Cognitive Functioning

In former football players, statistically significant associations were found between larger log-PVS and worse performance on the MoCA, a measure of general cognitive functioning (β = −0.74 [95% CI, −1.35 to −0.13]; *P* = .04; n = 159). We also found an association of larger log-PVS with worse performance on the Trail Making Test A, a measure of executive function (β = 2.78 [95% CI, 0.53 to 5.03]; *P* = .04; n = 159) (Table 2).

## Discussion

This cross-sectional study found that American football players had larger total WM PVS volume compared with an age-matched unexposed comparison group. Additionally, among former football players, larger PVS volume was associated with more extensive exposure to RHI, as well as with worse cognitive and executive functioning. These findings suggest an association between exposure to RHI, larger PVS volume, and worse cognitive function. This could indicate impaired brain clearance in former athletes with exposure to RHI.

Former football players had larger PVS volume, which is purported to reflect reduced perivascular transport.^30^ Moreover, within the group of former football players, larger PVS volume was associated with more extensive exposure to RHI. These findings are in line with studies investigating the effect of experimental brain injury on perivascular transport in rodents.^15^ However, the underlying pathomechanisms are not understood, although TBI-induced glial scarring may lead to impaired perivascular transport.^15,52^ More specifically, this glial scar is purported to reduce the polarization of aquaporin 4 channels, thereby potentially hindering perivascular fluid transport.^22,53,54^

We know less about perivascular transport in humans. There is initial evidence of an association between larger PVS volume and higher number of sustained mild TBIs in military veterans, another population at risk for neurodegeneration.^55^ Football players, too, are at increased risk for sustaining mild TBIs.^56^ However, most head impacts that occur in American football are likely subconcussive in nature, meaning they do not result in acute symptoms typically experienced in mild TBI.^57^ In this study, we found an association between PVS volume and estimated cumulative head impact force, but not with cumulative head impact frequency. PVS volume may thus be less influenced by sheer number of head impacts compared with the cumulative force of all impacts together.

MRI-visible PVS has long been believed to be a phenomenon in the context of normal brain aging.^58^ However, recent evidence points to a link between WM-PVS and cortical accumulation of neurotoxic metabolites in neurodegeneration.^32^ For example, a study by Perosa et al^32^ reported an association of antemortem MRI-visible WM-PVS and postmortem MRI-visible WM-PVS with the vascular deposition of amyloid beta within PVS in a sample of 19 participants with cerebral amyloid angiopathy and 5 controls. Perosa et al^32^ hypothesized that amyloid beta accumulation inside cortical vessel walls leads to an increase in vessel wall diameter, which in turn results in a thinning of the surrounding cortical PVS. This may cause an obstruction of the cortical PVS, resulting in distension of PVS in the adjacent WM.^59^ Another possible explanation for enlarged PVS is that RHI exposure leads to glial-vascular changes that reduce fluid and solute conductance from PVS into the surrounding interstitium.^24,60^ In this setting, ongoing perivascular CSF influx in the presence of reduced parenchymal conductance may lead to the dilation of the perivascular CSF flow channel observed in this study.

While either of these hypothesized models may explain the intramural accumulation of amyloid beta in cerebral amyloid angiopathy, in CTE, p-tau accumulation inside the vessel walls is not among the characteristic pathologies.^4,12,61–64^ In fact, neuropathological studies report tau accumulation to primarily occur in perivascular neurons, astrocytes, and oligodendrocytes.^4^ Several studies have reported that tau is cleared from the brain interstitium along perivascular compartments,^15,65^ and that brain injury impairs perivascular fluid exchange.^15,52^ Thus, we speculate that posttraumatic impairment of perivascular transport pathways results in both the distension of PVS and in the more rapid accumulation of intracellular p-tau pathology in perivascular regions. In the context of RHI exposure, the continuous posttraumatic release of intracellular tau and p-tau prior to the recovery of perivascular clearance may represent a particularly malignant pathological cascade and account for the known association between RHI and neurodegeneration.^66^

Of note, the association between RHI exposure and PVS was significantly modified by cardiovascular risk factors and medication. Interestingly, using β-blockers that cross the blood-brain barrier was associated with lower PVS volume, suggesting a possible protective association of this group of medications. Future research should further investigate the effect of β-blockers in the context of exposure to RHI.

Lastly, larger PVS volume was associated with worse global cognitive function and worse executive functioning in former football players even after adjusting for head impact exposure. To date, the literature on the association between PVS volume and neuropsychological functioning is equivocal. Depending upon the PVS quantification method chosen, MRI resolution, and population investigated, PVS measurements either do or do not show an association with cognitive function. We further discuss the existing literature in more detail in eAppendix 3 in Supplement 1. Of note, most studies using high-resolution MRI (as in this study) report associations between larger PVS volume and worse cognitive function.^67^ Findings from our study expand on these findings by revealing that larger PVS volume was particularly associated with executive function.

### Limitations

This study has some limitations. The cross-sectional design of this study limits interpretations. Thus, we need longitudinal or antemortem to postmortem comparisons to evaluate the prognostic value of PVS volume. Our findings also cannot be generalized beyond the current sample, as they are specific to former American football players who played in the 1970s to 2000s. Additionally, the calculated CHII scores are an estimation of head impacts received based on previously published accelerometer findings (in youth, high school, and college football), rather than participant-specific measurements. PVS volume does not directly measure cerebral fluid flux. Instead, PVS quantification serves as potential structural indicator of impaired fluid clearance.

## Conclusions

This cross-sectional study found that former American football players showed larger PVS volume compared with an unexposed comparison group. The more exposure to RHI, the larger the PVS volume in American football players. Larger PVS volume was also associated with worse overall cognitive and executive functioning. Taken together, these results suggest that enlarged PVS may indicate impaired brain clearance of neurotoxic waste products, which, in turn, are associated with neurodegenerative processes.

## Supplementary Material

Supplement 3 **eAppendix 1.** Processing, Perivascular Space Quantification, and Methodological Considerations

Supplement 2 **eAppendix 2.** Perivascular Space Volume, Demographics, and Medication

Supplement 1 **eAppendix 3.** Perivascular Space Volume and Cognitive Impairment
**eTable.** Perivascular Space Volume and Extensive Neuropsychological Assessments

## Figures and Tables

**Table 1.** Cohort Demographics

Characteristic	Participants, No. (%) (N = 224)
Age, median (IQR), y	57 (51–65)
BMI, median (IQR)	32 (29–35)
Education, median (IQR), y	16 (16–18)
Systolic blood pressure, median (IQR), mm Hg	129 (119–135)
Medication use^a^	
Antihypertensive	78 (35.1)
Blood-brain barrier permeable β-blocker	19 (8.6)
Statin	53 (23.9)
Antidiabetes medication	18 (8.1)
Hypercholesterolemia^a^	87 (39.2)
Diabetes^a^	18 (8.1)
*APOE* ε4 carrier^b^	58 (27.0)
Race and ethnicity	
American Indian or Alaska Native	1 (0.4)
Asian	0
Black or African American	75 (33.5)
Native Hawaiian or Pacific Islander	1 (0.4)
White	143 (63.8)
Multiple races	2 (0.9)
Did not report	2 (0.9)
Cognitive functioning score, median (IQR)^c^	
Montreal Cognitive Assessment	26 (24–27)
NAB List Learning Test	5 (4–8)
Trail Making Test A	27.3 (22.5–35.4)
Trail Making Test B	69.0 (52.4–93.5)
Golden Stroop Color-Word Interference, mean (SD)	37.0 (9.5)
Perivascular Space	
WM volume, mean (SD), cm^3^	470.3 (78.5)
WM-PVS volume, median (IQR), mm^3^	4063 (2512–6272)
Log-WM-PVS fraction, mean (SD)	0.0 (1.0)
Exposure to RHI^d^	
Duration of football play, mean (SD), y	15.9 (4.3)
Age of first exposure, median (IQR), y	11.5 (9.0–13.3)
CHII frequency, median (IQR), impacts × 10^4^	9.8 (7.7–13.4)
CHII linear force, median (IQR), g-force × 10^6^	2.2 (1.8–2.7)
CHII rotational force, median (IQR), radians/s^2^ × 10^8^	1.8 (1.4–2.2)
Position group at highest level of play	
Offensive linemen	41 (24.1)
Offensive backs and receivers	48 (28.2)
Defensive linemen	19 (11.1)
Linebackers	26 (15.3)
Defensive backs	33 (19.4)
Special teams	3 (1.8)

Abbreviations: NAB, neuropsychological assessment battery; PVS, perivascular space; WM, white matter.

a Data on hypercholesterolemia, diabetes, statin use, antihypertensive medication use, and blood-brain barrier permeable β-blocker use were available for 222 participants.

b *APOE* ε4-carrier analysis was available for 215 participants.

c The Montreal Cognitive Assessment and the Trail Making Test A and B were taken by 223 participants. The NAB List Learning Test long delay recall was performed by 222 participants, and the Golden Stroop color word interference by 220 participants.

d Measured only among former American football players (n = 170).

**Table 2.** Association of Perivascular Space Volume and Neuropsychological Functioning

Neuropsychological evaluation	β [95%CI]	*P* value^a^
Montreal Cognitive Assessment (n = 159)	−0.74 (−1.35 to −0.13)	.04
NAB List Learning Test (n = 159)	−0.21 (−0.77 to 0.35)	.46
Trail Making Test A (n = 159)	2.78 (0.53 to 5.03)	.04
Trail Making Test B (n = 159)	6.01 (−2.05 to 14.08)	.24
Golden Stroop Color-Word Interference (n = 157)^b^	−1.03 (−2.73 to 0.67)	.29

Abbreviation: NAB, neuropsychological assessment battery.

a Multiple generalized linear regression models were used to analyze the association of log perivascular space with neuropsychological tests. The reduced number of participants included in the analysis is due to nonavailable covariates.

b The Golden Stroop Color Word Interference was only performed by 167 former football player participants.

## Data Availability

See Supplement 3.

## Key Points

### Question

Is perivascular space (PVS) volume associated with repetitive head impacts (RHI) in former American football players at risk for neurodegeneration?

### Findings

In this cross-sectional cohort study of 170 former American football players and 54 unexposed controls, larger PVS volume was associated with greater exposure to RHI. Additionally, PVS volume was associated with worse performance on cognitive tests.

### Meaning

These findings suggest that PVS volume may contribute to the association between exposure to RHI, cognitive impairment, and the development of RHI-associated neurodegeneration.
