# Supplementary material for: Repetitive Head Impacts and Perivascular Space Volume in Former American Football Players
Source: JAMA Netw Open. Author manuscript; Available in PMC 2025 Aug 1. (PMC12025916; doi:10.1001/jamanetworkopen.2024.28687)
Supplement: Supplement 3 — eAppendix 1. Processing, Perivascular Space Quantification, and Methodological Considerations [file NIHMS2044232-supplement-Supplement_3.pdf]

Data Sharing Statement

Jung. Repetitive Head Impacts and Perivascular Space Volume in Former American Football Players. *JAMA Netw Open*. Published August 16, 2024.
doi:10.1001/jamanetworkopen.2024.28687

Data
Data available: Yes
Data types: Deidentified participant data, Data dictionary
How to access data: The datasets generated and analyzed during the current study will be available in the Federal Interagency Traumatic Brain Injury Research (FITBIR) repository, https://fitbir.nih.gov. Datasets will also be available through a data-sharing portal for the DIAGNOSE CTE Research Project, http://diagnosecte.com. It is also anticipated that study datasets will be available in the Global Alzheimer's Association Interactive Network (GAAIN) repository, http://www.gaain.org.
When available: beginning date: 10-01-2025

Supporting Documents
Document types: None

Additional Information
Who can access the data: researcher whose proposed use of the data has been approved
Types of analyses: for research purposes
Mechanisms of data availability: after approval of a proposal
