# Supplementary material for: Repetitive Head Impacts and Perivascular Space Volume in Former American Football Players
Source: JAMA Netw Open. Author manuscript; Available in PMC 2025 Aug 1. (PMC12025916; doi:10.1001/jamanetworkopen.2024.28687)
Supplement: Supplement 2 — eAppendix 2. Perivascular Space Volume, Demographics, and Medication [file NIHMS2044232-supplement-Supplement_2.xlsx]

Supplemental Online Content: Nonauthor Collaborators
*First name, last name, and suffix (if applicable) are required and will appear in PubMed.

*Group Name(s): DIAGNOSE CTE Research Project Study Team

*First Name and Middle Initial(s)	*Last Name	*Suffix (eg, Jr, III)	Academic Degrees	Institution	Location (city, state/province, country)	Role or Contribution, eg, chair, principal investigator	Group (if more than 1 Group listed in the byline) and/or Subgroup (eg, Steering Committee)
Yi	Su		Ph.D.	Banner Alzheimer's Institute	Phoenix, Arizona, US	contributor	
Kewei	Chen		Ph.D.	Banner Alzheimer's Institute	Phoenix, Arizona, US	contributor	
Hillary	Protas		Ph.D.	Banner Alzheimer's Institute	Phoenix, Arizona, US	contributor	
Conni	Boker		M.B.A.	Banner Alzheimer's Institute	Phoenix, Arizona, US	contributor	
Rhoda	Au		Ph.D.	Boston University School of Medicine	Boston, Massachusetts, US	contributor	
Robert C.	Cantu		Ph.D.	Boston University School of Medicine	Boston, Massachusetts, US	contributor	
Lindsay	Farrer		Ph.D.	Boston University School of Medicine	Boston, Massachusetts, US	contributor	
Robert	Helm		M.D.	Boston University School of Medicine	Boston, Massachusetts, US	contributor	
Neil	Kowall		M.D.	Boston University School of Medicine	Boston, Massachusetts, US	contributor	
Gustavo	Mercier		M.D., Ph.D.	Boston University School of Medicine	Boston, Massachusetts, US	contributor	
James	Otis		M.D.	Boston University School of Medicine	Boston, Massachusetts, US	contributor	
Jason	Weller		M.D.	Boston University School of Medicine	Boston, Massachusetts, US	contributor	
Irene	Simkin		M.S.	Boston University School of Medicine	Boston, Massachusetts, US	contributor	
Alondra	Andino		B.A.	Boston University Project Coordinating Center Staff	Boston, Massachusetts, US	coordination	
Shannon	Conneely		B.A.	Boston University Project Coordinating Center Staff	Boston, Massachusetts, US	coordination	
Courtney	Diamond		M.B.A.	Boston University Project Coordinating Center Staff	Boston, Massachusetts, US	coordination	

Supplemental Online Content: Nonauthor Collaborators
*First name, last name, and suffix (if applicable) are required and will appear in PubMed.

***First Name and Middle Initial(s)**	***Last Name**	***Suffix (eg, Jr, III)**	Academic Degrees	Institution	Location (city, state/province, country)	Role or Contribution, eg, chair, principal investigator	Group (if more than 1 Group listed in the byline) and/or Subgroup (eg, Steering Committee)
Tessa	Fagle		B.A.	Boston University Project Coordinating Center Staff	Boston, Massachusetts, US	coordination	
Olivia	Haller		B.A.	Boston University Project Coordinating Center Staff	Boston, Massachusetts, US	coordination	
Tennyson	Hung		M.B.A.	Boston University Project Coordinating Center Staff	Boston, Massachusetts, US	coordination	
Nicole	Gullotti		M.B.A.	Boston University Project Coordinating Center Staff	Boston, Massachusetts, US	coordination	
Megan	Mariani		B.S., B.A.	Boston University Project Coordinating Center Staff	Boston, Massachusetts, US	coordination	
Brian	Mayville		B.S., B.A.	Boston University Project Coordinating Center Staff	Boston, Massachusetts, US	coordination	
Kathleen	McLaughlin		B.A.	Boston University Project Coordinating Center Staff	Boston, Massachusetts, US	coordination	
Mary	Nanna		B.A.	Boston University Project Coordinating Center Staff	Boston, Massachusetts, US	coordination	
Taylor	Platt		M.P.H.	Boston University Project Coordinating Center Staff	Boston, Massachusetts, US	coordination	
Sury	Pulukuri		B.A.	Boston University Project Coordinating Center Staff	Boston, Massachusetts, US	coordination	
Fiona	Rica		M.P.H.	Boston University Project Coordinating Center Staff	Boston, Massachusetts, US	coordination	
Madison	Sestak		B.S.	Boston University Project Coordinating Center Staff	Boston, Massachusetts, US	coordination	
Michael	McClean		Sc.D.	Boston University School of Public Health	Boston, Massachusetts, US	contributor	
Douglas	Annis		M.S.	Boston University School of Public Health	Boston, Massachusetts, US	contributor	
Christine	Chaisson		M.P.H.	Boston University School of Public Health	Boston, Massachusetts, US	contributor	

Supplemental Online Content: Nonauthor Collaborators
*First name, last name, and suffix (if applicable) are required and will appear in PubMed.

***First Name and Middle Initial(s)**	***Last Name**	***Suffix (eg, Jr, III)**	Academic Degrees	Institution	Location (city, state/province, country)	Role or Contribution, eg, chair, principal investigator	Group (if more than 1 Group listed in the byline) and/or Subgroup (eg, Steering Committee)
Dianne B.	Dixon			Boston University School of Public Health	Boston, Massachusetts, US	contributor	
Carolyn	Finney		B.A.	Boston University School of Public Health	Boston, Massachusetts, US	contributor	
Kerrin	Gallagher		M.P.H	Boston University School of Public Health	Boston, Massachusetts, US	contributor	
Kaitlin	Hartlage		M.P.H	Boston University School of Public Health	Boston, Massachusetts, US	contributor	
Jun	Lu		M.S.	Boston University School of Public Health	Boston, Massachusetts, US	contributor	
Brett	Martin		M.S.	Boston University School of Public Health	Boston, Massachusetts, US	contributor	
Emmanuel	Ojo		M.P.H.	Boston University School of Public Health	Boston, Massachusetts, US	contributor	
Joseph N.	Palmisano		M.A., M.P.H.	Boston University School of Public Health	Boston, Massachusetts, US	contributor	
Brittany	Pine		B.A., B.S.	Boston University School of Public Health	Boston, Massachusetts, US	contributor	
Janani	Ramachandran		M.S.	Boston University School of Public Health	Boston, Massachusetts, US	contributor	
Jennifer	Fitzsimmons		M.D.	Brigham and Women's Hospital	Boston, Massachusetts, US	contributor	
Elena M.	Bonke		Ph.D.	Brigham and Women's Hospital	Boston, Massachusetts, US	contributor	
Katherine	Breedlove		Ph.D.	Brigham and Women's Hospital	Boston, Massachusetts, US	contributor	
Eduardo	Coello		Ph.D.	Brigham and Women's Hospital	Boston, Massachusetts, US	contributor	
Hujiun	Liao		B.S.	Brigham and Women's Hospital	Boston, Massachusetts, US	contributor	

Supplemental Online Content: Nonauthor Collaborators
*First name, last name, and suffix (if applicable) are required and will appear in PubMed.

***First Name and Middle Initial(s)**	***Last Name**	***Suffix (eg, Jr, III)**	Academic Degrees	Institution	Location (city, state/province, country)	Role or Contribution, eg, chair, principal investigator	Group (if more than 1 Group listed in the byline) and/or Subgroup (eg, Steering Committee)
Maria Loy			M.B.A., M.P.H.	Brigham and Women's Hospital	Boston, Massachusetts, US	contributor	
Elizabeth	Rizzoni		B.A.	Brigham and Women's Hospital	Boston, Massachusetts, US	contributor	
Vivian	Schultz		M.D.	Brigham and Women's Hospital	Boston, Massachusetts, US	contributor	
Annelise	Silva		B.S.	Brigham and Women's Hospital	Boston, Massachusetts, US	contributor	
Brynn	Vessey		B.S.	Brigham and Women's Hospital	Boston, Massachusetts, US	contributor	
Sarah	Banks		Ph.D.	Cleveland Clinic Lou Ruvo Center for Brain Health	Las Vegas, Nevada, US	contributor	
Jason	Miller		Ph.D.	Cleveland Clinic Lou Ruvo Center for Brain Health	Las Vegas, Nevada, US	contributor	
Aaron	Ritter		M.D.	Cleveland Clinic Lou Ruvo Center for Brain Health	Las Vegas, Nevada, US	contributor	
marwan	Sabbagh		M.D.	Cleveland Clinic Lou Ruvo Center for Brain Health	Las Vegas, Nevada, US	contributor	
Raelynn	de la Cruz			Cleveland Clinic Lou Ruvo Center for Brain Health	Las Vegas, Nevada, US	contributor	
Jan	Durant			Cleveland Clinic Lou Ruvo Center for Brain Health	Las Vegas, Nevada, US	contributor	
Moragn	Golceker			Cleveland Clinic Lou Ruvo Center for Brain Health	Las Vegas, Nevada, US	contributor	
Nicolette	Harmon			Cleveland Clinic Lou Ruvo Center for Brain Health	Las Vegas, Nevada, US	contributor	
Kaeson	Kaylegian			Cleveland Clinic Lou Ruvo Center for Brain Health	Las Vegas, Nevada, US	contributor	
Rachelle	Long			Cleveland Clinic Lou Ruvo Center for Brain Health	Las Vegas, Nevada, US	contributor	

Supplemental Online Content: Nonauthor Collaborators
*First name, last name, and suffix (if applicable) are required and will appear in PubMed.

***First Name and Middle Initial(s)**	***Last Name**	***Suffix (eg, Jr, III)**	Academic Degrees	Institution	Location (city, state/province, country)	Role or Contribution, eg, chair, principal investigator	Group (if more than 1 Group listed in the byline) and/or Subgroup (eg, Steering Committee)
Christin	Nance			Cleveland Clinic Lou Ruvo Center for Brain Health	Las Vegas, Nevada, US	contributor	
Priscilla	Sandoval			Cleveland Clinic Lou Ruvo Center for Brain Health	Las Vegas, Nevada, US	contributor	
Robert W.	Turner	III	Ph.D.	George Washington University School of Medicine and Health Sciences	Washington, D.C., US	contributor	
Kenneth L.	Marek		M.D.	Invicro (formerly Molecular NeuroImaging)	Needham, Massachusetts, US	contributor	
Andrew	Serrano		M.B.A.	Invicro (formerly Molecular NeuroImaging)	Needham, Massachusetts, US	contributor	
David W.	Dodick		M.D.	Mayo Clinic Arizona	Phoenix, Arizona, US	contributor	
Yonas	Geda		M.D., MSc	Mayo Clinic Arizona	Phoenix, Arizona, US	contributor	
Jennifer V.	Wethe		Ph.D.	Mayo Clinic Arizona	Phoenix, Arizona, US	contributor	
Bryce	Falk		R.N.	Mayo Clinic Arizona	Phoenix, Arizona, US	contributor	
Amy	Duffy			Mayo Clinic Arizona	Phoenix, Arizona, US	contributor	
Marci	Howard			Mayo Clinic Arizona	Phoenix, Arizona, US	contributor	
Michelle	Montague			Mayo Clinic Arizona	Phoenix, Arizona, US	contributor	
Thomas	Osgood			Mayo Clinic Arizona	Phoenix, Arizona, US	contributor	
Debra	Babcock		M.D., Ph.D.	National Institute of Neurological Disorders and Stroke (NINDS)	Bethesda, Maryland, US	Administrative Program Official	
William	Barr		Ph.D.	New York University	New York, New York, US	contributor	
Judith	Goldberg		Sc.D.	New York University	New York, New York, US	contributor	
Thomas	Wisniewski		M.D.	New York University	New York, New York, US	contributor	
Ivian	Kirov		Ph.D.	New York University	New York, New York, US	contributor	
Yvonne	Lui		M.D.	New York University	New York, New York, US	contributor	
Charles	Marmar		M.D.	New York University	New York, New York, US	contributor	
Lisene	Hasanaj			New York University	New York, New York, US	contributor	
Liliana	Serrano			New York University	New York, New York, US	contributor	
Alhassan	Al-Kharafi			New York University	New York, New York, US	contributor	

Supplemental Online Content: Nonauthor Collaborators
*First name, last name, and suffix (if applicable) are required and will appear in PubMed.

***First Name and Middle Initial(s)**	***Last Name**	***Suffix (eg, Jr, III)**	Academic Degrees	Institution	Location (city, state/province, country)	Role or Contribution, eg, chair, principal investigator	Group (if more than 1 Group listed in the byline) and/or Subgroup (eg, Steering Committee)
Allan	George			New York University	New York, New York, US	contributor	
Sammie	Martin			New York University	New York, New York, US	contributor	
Edward	Riley			New York University	New York, New York, US	contributor	
William	Runge			New York University	New York, New York, US	contributor	
Elaine R.	Peskind		M.D.	University of Washington and VA Puget Sound	Seattle, Washington, US	contributor	
Elizabeth	Colasurdo			University of Washington and VA Puget Sound	Seattle, Washington, US	contributor	
Daniel S.	Marcus		Ph.D.	Washington University (CNDA)	St. Louis, Missouri, US	contributor	
Jenny	Gurney		M.S.	Washington University (CNDA)	St. Louis, Missouri, US	contributor	
Keith A.	Johnson		M.D.	Massachusetts General Hospital	Boston, Massachusetts, US	contributor	
Richard	Greenwald		Ph.D.	Simbex	Lebanon, New Hampshire, US	contributor	
