# Supplementary material for: Repetitive Head Impacts and Perivascular Space Volume in Former American Football Players
Source: JAMA Netw Open. Author manuscript; Available in PMC 2025 Aug 1. (PMC12025916; doi:10.1001/jamanetworkopen.2024.28687)
Supplement: Supplement 1 — eAppendix 3. Perivascular Space Volume and Cognitive Impairment eTable. Perivascular Space Volume and Extensive Neuropsychological Assessments [file NIHMS2044232-supplement-Supplement_1.pdf]

Supplemental Online Content

Jung LB, Wiegand TLT, Tuz-Zahra F, et al; DIAGNOSE CTE Research Project. Repetitive head impacts and perivascular space volume in former American football players. *JAMA Netw Open*. 2024;7(8):e2428687. doi:10.1001/jamanetworkopen.2024.28687

eAppendix 1. Processing, Perivascular Space Quantification, and Methodological Considerations
eAppendix 2. Perivascular Space Volume, Demographics, and Medication
eAppendix 3. Perivascular Space Volume and Cognitive Impairment
eTable. Perivascular Space Volume and Extensive Neuropsychological Assessments
eReferences.

This supplemental material has been provided by the authors to give readers additional information about their work.

© 2024 Jung LB et al. *JAMA Network Open.*

eAppendix 1

Processing & Perivascular Space Quantification

For multi-step MRI-image processing the Psychiatry Neuroimaging Laboratory luigi pipeline (https://github.com/pnlbwh/luigi-pnlpipe) was used. First, the acquired raw data were converted from dicom to nifti format. Next, the nifti files were axis-aligned and centered. Brain-masks were created using the luigi-pnlpipe StructMask module. The images were then visually inspected for completeness and artefacts (e.g., motion). The masks were manually corrected to perfectly fit individual brain size, using 3D Slicer (http://www.slicer.org; version 4.5, Surgical Planning Laboratory, Brigham and Women's Hospital, Boston, MA, USA). Automated brain segmentation was performed using the "recon-all" module of FreeSurfer (version 7.1.0 https://surfer.nmr.mgh.harvard.edu, Laboratory for Computational Neuroimaging, Charlestown, MA, USA) with the Desikan-Killiany atlas as a template.[1]

The perivascular space (PVS) volume was quantified using a previously published method by Sepehrband et al..[2] To remove non-structured high-frequency spatial noise from T1w- and T2w- images, we used the non-local mean filtering technique.[3] This technique measures the similarities of image intensities, while considering the neighboring voxels using a blockwise approach. A 1-voxel radius filtering patch was applied. This removes noise at a single voxel level but keeps spatially repeated signal intensities and thus preserves PVS. Finally, by dividing the filtered T1w- image by the filtered T2w- image, an enhanced perivascular space contrast (EPC) image was derived.

Parcellated white matter (WM) was created using the Advanced Normalization Tool packages (ANT-packages) (http://stnava.github.io/ANTs/) n-tissue parcellation technique to be later used as a WM-mask. Utilizing the Quantitative Imaging Toolkit (QIT) (https://cabeen.io/qitwiki/), we applied a Frangi filter to the EPC. The Frangi filter derives a vesselness likelihood for each voxel from the eigenvector of the images Hessian matrix to approximate the likelihood of a voxel being part of a vessel-like structure in the MRI-image. We used the recommended default parameters (α = 0.5, β = 0.5 and c set to ½ the value of the Hessian norm).[4] To maximize vessel inclusion, the range was set at 0.1 – 5 voxels. A vesselness threshold of 0.9e-9 best fit the study data and was thus used to obtain the final WM-PVS mask. Every WM-PVS mask was evaluated and manually corrected by an experienced rater. To assess intra-rater reliability, the rater re-assessed 10 random WM-PVS masks (>10% of all WM-PVS masks), 2 weeks after the initial evaluation. An intra-class correlation coefficient was calculated (single rater, 2-way-mixed-effects model, absolute agreement), resulting in an intra-class correlation coefficient (ICC) of *>0.99, P<.001.*

However, 10 former football players were excluded due to MRI acquisition or processing errors [T1-weighted (T1w) images missing (n=6); T1w images failed FreeSurfer processing (n=1); failed PVS processing (n=1); T2-weighted (T2w) images missing (n=1); scanned using another MRI scanner with different sequence parameters (n=1)]. Additionally, data of 6 unexposed participants were excluded [missing T1w images (n=1), missing T2w images (n=1), undisclosed exposure to RHI (n=3), undisclosed long-standing psychiatric disorder (n=1)], resulting in 54 unexposed participants.

Methodological Considerations

PVS are fluid filled compartments surrounding the cerebral vessels. Physiologically, PVS are considered to be sub-millimeter in diameter.[5] However, PVS in the WM can increase in diameter and can therefore become visible on non-contrast MRI.[6] Of note, a recent study reports in-vivo PVS (MRI) to be highly associated with post-mortem PVS morphology (histopathological evaluation),[6] thus supporting the use of non-contrast MRI as a way of defining PVS properties.

Most previous studies have assessed PVS using visual rating scales that are applied to anatomical sequences (i.e., T1w or FLAIR). Raters count visible PVS manually within specific anatomical regions (e.g., the centrum semiovale[7]) on a single MRI slice to then estimate the overall PVS load.[8] Visual rating scales thus do not use specific software but are easy to apply. However, underlying subjective bias may be present and there may be a loss of sensitivity as such measures do not cover the heterogeneity of PVS across brain regions. Fortunately, advances in MRI resolution and computational image processing over the last decade have led to more recent studies using image processing algorithms to quantify PVS volume more objectively and across all brain slices, which is not possible with labor intensive manual measures.[2] Thus by using such processing algorithms for the quantification of PVS volume across the entire brain, the heterogeneity of individual PVS morphology can be sensitively captured. Moreover, compared to visual rating scales, computational PVS quantification techniques are far less dependent upon rater bias. Although compared to standard visual rating scales, computational PVS quantification likely provides a more accurate measurement of PVS volume, it should be noted that image resolution of 1x1x1mm resolution at 3T nonetheless limits the quantification of microscopic PVS morphology.

© 2024 Jung LB et al. *JAMA Network Open.*

eAppendix 2

Perivascular Space Volume, Demographics and Medication

All information on medical conditions and treatment reported in this study are based on self-report. Out of the 227 participants, 225 answered yes/no questions regarding whether they were receiving treatment for specific relevant cardiovascular diseases (e.g., hypertension, hypercholesterolemia, and/or diabetes). Among these respondents, 216 participants (95 %) provided detailed information about their medication intake. To ensure a larger sample size, participants who did not state their detailed medical information, but were not undergoing treatment for relevant cardiovascular diseases, were also included.

To compare differences in log-PVS between former football players taking blood brain barrier permeable β-blockers and those without, an ANCOVA was performed. Results showed log-PVS was statistically significantly *reduced* in former football players taking a blood brain barrier permeable β-blocker, even though the analysis was also adjusted for general intake of anti-hypertensive medication (*mean difference=.62 [95%CI .11-1.13]; P=.02; n=160*). Larger PVS volume is thus observed in football players with *general anti-hypertensive* medication, possibly due to the underlying hypertensive disease. Importantly, while the associations between higher age and larger PVS volume have been reported before[9,10], lower PVS volume in those taking β-blockers has not.

Prior studies in animals have focused on the role of the noradrenergic system in brain clearance[11,12]. It is hypothesized that α_1-antagonists and β-blockers inhibit the release of norepinephrine, which possibly reduces interstitial resistance and therefore enhances perivascular clearance[11,12]. Although we provide initial evidence of β-blocker intake being associated with possible imaging markers of neurodegeneration, longitudinal studies are needed to investigate the role of β-blocker intake on PVS volume and long-term outcome in populations exposed to RHI who are at risk for neurodegenerative diseases.

© 2024 Jung LB et al. *JAMA Network Open.*

eAppendix 3

Perivascular Space Volume and Cognitive Impairment

The association between PVS volume and cognitive impairment is still not entirely clear. Vast improvements in MRI resolution and PVS quantification software make study results difficult to compare with those conducted prior to the advent of imaging studies. Still, Paradise et al.[7] acquired 414 structural 3T MRI scans of community-dwelling adults and counted the number of visible PVS in a single slice that depicted the centrum semiovale. In this longitudinal study, participants within the top quartile regarding number of PVS were more likely to develop MCI 4 years later and dementia 8 years later. On the other hand, a meta-analysis by Hilal et al.[13] which included 5 population-based studies totaling 3575 participants aged 60-90 years old, did not find an association between the number of PVS larger than 1mm and the mini mental state examination. Of note, comparing the above studies to the current study, there are considerable methodological differences. Specifically, Hilal et al. [13] used a visual rating scale instead of a computational quantification of PVS volume. Further, more than 65 percent of the MRI scans included in the study by Hilal et al. were acquired on a 1.5T scanner. The lower image resolution of the 1.5T scanner, combined with the visual identification of PVS, likely resulted in a lack of sensitivity to quantifying PVS in this population. Accordingly, further evidence is needed using uniform study populations, imaging methods, and PVS quantification.

Nonetheless, most studies using novel PVS quantification algorithms on high-resolution 3T MRI report associations between larger PVS volume and worse performance on neuropsychological tests. Therefore, PVS volume may be a sensitive imaging marker for cognitive dysfunction in the context of neurodegeneration.

© 2024 Jung LB et al. *JAMA Network Open.*

eTable 1
Perivascular Space Volume and Extensive Neuropsychological Assessments

Neuropsychological Evaluation[a]	β-value [95%CI]
Unified Data Set Multilingual Naming Test (n=159)	-.01 [-.49 – .47]
Category Semantic Fluency – Animals (n=159)	-.08 [-1.04 – .88]
Brief Visuospatial Memory Test – Revised: Copy Task (n=158)	-.11 [-.23 – .01]
Judgment of Line Orientation (n=159)	-.22 [-.64 – .20]
Neuropsychological Assessment Battery – Mazes (n=159)	-1.46 [-2.55 – -.37]
Behavior Rating Inventory of Executive Function-A Metacognition Index (n=160)	.55 [-3.02 – .32]
Number Span – forward (n=158)	-.04 [-.46 – .38]
Number Span – backward (n=158)	-.01 [-.44 – .43]
Montreal Cognitive Assessment (MoCA) (n=159)	
MoCA – Visuospatial	-.23 [-.39 – -.06]
MoCA – Naming	.02 [-.06 – .10]
MoCA – Attention	-.16 [-.33 – -.00]
MoCA – Language	-.03 [-.19 – .13]
MoCA – Abstraction	.02 [-.10 – .13]
MoCA – Delayed Recall	-.24 [-.52 – .04]
MoCA – Orientation	-.14 [-.26 – -.01]

NOTE:
[a] Multiple generalized linear regression models were used to associate log-PVS with an extended battery of neuropsychological assessments in former American football players. The regression coefficients and 95% confidence intervals are reported as β-values and 95%CI. The neuropsychological tests included: 1) language ability (Unified Data Set Multilingual Naming Test; Category Semantic Fluency – Animals), 2) visuospatial ability (Brief Visuospatial Memory Test – Revised: Copy Task; Judgement of Line Orientation), 3) executive function (Neuropsychological assessment battery – Maze Test; Behavior Rating Inventory of Executive Function-A Metacognition Index), 4) memory (Number Span forward and backward test), and 5) Montreal Cognitive Assessment (MoCA) including its sub scores: Visuospatial, Naming, Attention, Language, Abstraction, Delayed Memory, Orientation. All analyses are corrected for the co-variates detailed in the main manuscript.
The Behavior Rating Inventory of Executive Function-A Metacognition Index was complete by all participants. The Brief Visuospatial Memory Test – Revised: Copy Task and the Number Span tests were only performed by 168, while other tests were performed by 169 former American football players. The reduced number of subjects included in the analysis is due to non-available co-variates.

© 2024 Jung LB et al. *JAMA Network Open.*

eReferences.

1. Desikan RS, Segonne F, Fischl B, et al. An automated labeling system for subdividing the human cerebral cortex on MRI scans into gyral based regions of interest. *Neuroimage.* 2006;31(3):968-980.
2. Sepehrband F, Barisano G, Sheikh-Bahaei N, et al. Image processing approaches to enhance perivascular space visibility and quantification using MRI. *Sci Rep.* 2019;9(1):12351.
3. Manjon JV, Coupe P, Marti-Bonmati L, Collins DL, Robles M. Adaptive non-local means denoising of MR images with spatially varying noise levels. *J Magn Reson Imaging.* 2010;31(1):192-203.
4. Frangi AF, Niessen, W. J., Vincken, K. L. & Viergever, M. A. Multiscale vessel enhancement filtering. *International Conference on Medical Image Computing and Computer-Assisted Intervention.* 1998;130–137.
5. Abbott NJ, Pizzo ME, Preston JE, Janigro D, Thorne RG. The role of brain barriers in fluid movement in the CNS: is there a 'glymphatic' system? *Acta Neuropathol.* 2018;135(3):387-407.
6. Perosa V, Oltmer J, Munting LP, et al. Perivascular space dilation is associated with vascular amyloid-beta accumulation in the overlying cortex. *Acta Neuropathol.* 2022;143(3):331-348.
7. Paradise M, Crawford JD, Lam BCP, et al. Association of Dilated Perivascular Spaces With Cognitive Decline and Incident Dementia. *Neurology.* 2021;96(11):e1501-e1511.
8. Paradise MB, Beaudoin MS, Dawes L, et al. Development and validation of a rating scale for perivascular spaces on 3T MRI. *J Neurol Sci.* 2020;409:116621.
9. Lynch KM, Sepehrband F, Toga AW, Choupan J. Brain perivascular space imaging across the human lifespan. *Neuroimage.* 2023;271:120009.
10. Pinheiro A, Demissie S, Scruton A, et al. Association of Apolipoprotein E varepsilon4 Allele with Enlarged Perivascular Spaces. *Ann Neurol.* 2022;92(1):23-31.
11. Xie L, Kang H, Xu Q, et al. Sleep drives metabolite clearance from the adult brain. *Science.* 2013;342(6156):373-377.
12. Lilius TO, Blomqvist K, Hauglund NL, et al. Dexmedetomidine enhances glymphatic brain delivery of intrathecally administered drugs. *J Control Release.* 2019;304:29-38.
13. Hilal S, Tan CS, Adams HHH, et al. Enlarged perivascular spaces and cognition: A meta-analysis of 5 population-based studies. *Neurology.* 2018;91(9):e832-e842.

© 2024 Jung LB et al. *JAMA Network Open.*
